# Modelling the effect of subcellular mutations on the migration of cells in the colorectal crypt

**DOI:** 10.1186/s12859-020-3391-3

**Published:** 2020-03-03

**Authors:** Lotte B. Romijn, Axel A. Almet, Chin Wee Tan, James M. Osborne

**Affiliations:** 1 School of Mathematics and Statistics, University of Melbourne, Parkville, VIC Australia; 2 Wolfson Centre for Mathematical Biology, Mathematical Institute, University of Oxford, Oxford, UK; 3 Personalised Oncology Division, The Walter and Eliza Hall Institute of Medical Research, Parkville, VIC Australia; 4 Department of Medical Biology, University of Melbourne, Parkville, VIC Australia; 5 NSF-Simons Center for Multiscale Cell Fate Research, University of California, Irvine, California USA; 6 Department of Mathematics, University of California, Irvine, California USA

**Keywords:** Colorectal cancer, Crypt, Multicellular modelling, SBML, Chaste

## Abstract

**Background:**

Many cancers arise from mutations in cells within epithelial tissues. Mutations manifesting at the subcellular level influence the structure and function of the tissue resulting in cancer. Previous work has proposed how cell level properties can lead to mutant cell invasion, but has not incorporated detailed subcellular modelling

**Results:**

We present a framework that allows the straightforward integration and simulation of SBML representations of subcellular dynamics within multiscale models of epithelial tissues. This allows us to investigate the effect of mutations in subcellular pathways on the migration of cells within the colorectal crypt. Using multiple models we find that mutations in APC, a key component in the Wnt signalling pathway, can bias neutral drift and can also cause downward invasion of mutant cells in the crypt.

**Conclusions:**

Our framework allows us to investigate how subcellular mutations, i.e. knockouts and knockdowns, affect cell-level properties and the resultant migration of cells within epithelial tissues. In the context of the colorectal crypt, we see that mutations in APC can lead directly to mutant cell invasion.

## Background

Colorectal cancer (CRC) is one of the most common forms of cancer and a leading cause of cancer death in the western world [1]. CRC’s initiation has been attributed to a loss of control over the proliferation and migration of colon crypt cells [2, 3] with cell proliferation and crypt production processes playing crucial roles in the growth of colorectal adenomas and hyperplastic polyps [4]. The Wnt signalling pathway has been linked to intestinal crypt formation, cell proliferation [5–7] and the regulation of cell-cell adhesion in crypts [8, 9]. With 90% of CRCs having mutations in key components of the Wnt pathway, i.e. adenomatous polyposis coli (APC) or *β*-catenin (*β*-cat) [10], it is inevitable that this pathway attracts considerable attention. Oddly, the importance of cell-cell adhesion’s association with *β*-cat has often being overlooked, particularly as E-cadherin (E-cad) has several critical functions, including facilitating the normal homeostasis and morphogenesis of the intestine [11, 12], serving as a tumor suppressor gene [13] and preventing invasiveness in carcinomas cells [14]. Currently, neither the roles of Wnt signalling and cell-cell adhesion in the colon nor the links between the underlying APC, *β*-catenin and E-cadherin biochemistry and adenoma formation are well understood.

The Wnt/ *β*-catenin pathway has been commonly known to regulate the multi-functional protein *β*-catenin’s concentration within the cell [15, 16]. In the absence of Wnt signaling, *β*-catenin forms a complex with the scaffold proteins Axin and APC to form a complex known as the degradation complex [17, 18]. This degradation complex facilitates the phosphorylation of *β*-catenin by glycogen synthase kinase-3- *β* (GSK3 *β*) [19], which targets phosphorylated *β*-catenin for degradation via the proteasome pathway [20]. Wnt ligands activates the Wnt pathway bringing about the disruption of the degradation complex and consequently reduction in *β*-catenin degradation within the cytosol [19, 21, 22]. The subsequent increase in *β*-catenin concentrations lead to the formation of a downstream complex *β*-catenin: T-Cell Factor complex (TCF, a transcription co-factor) in the nucleus. The *β*-catenin-TCF complex formation activates gene transcriptional activities promoting cellular functions like cell proliferation, survival and cell fate decisions [23–25]. Another major function of *β*-catenin is in cell-cell adhesion, with the Cadherin family proteins serving as key adhesion partners of *β*-catenin in the cell-cell adhesion processes [26, 27]. These interactions will influence the distribution of *β*-catenin within the cell. Mutation in the tumor suppressor gene APC accounts for most CRC cases. As a key scaffolding protein of the degradation complex, its mutation causes an inability to form the complex and subsequently leads to the stabilization of *β*-catenin in the cell. This leads to downstream gene transcription and consequently affects cell-adhesion and migration [28].

### Multicellular simulations of the crypt

The crypt has been the focus of numerous multicellular modelling studies, many of which have been discussed in literature reviews such as Kershaw et al. [29]. The work most relevant to this study is by Van Leeuwen et al. [30], who incorporate a Wnt-dependent subcellular model within the off-lattice, cell-centre model for tissue mechanics by Meineke et al. [31]. Wnt signalling is modelled to affect both proliferative capacity and adhesion, as based on previous work by the authors [32]. The framework is used to investigate clonal expansion of cell lineages and the effect of Wnt signalling on spatial variation in nuclear *β*-catenin concentrations. We will now discuss the features of this work that are pertinent to our study.

In Van Leeuwen et al. [30], the crypt is modelled as a rolled-out cylinder in 2D. This geometry has become a popular candidate for modelling the crypt, though other geometries have been considered [33–36]. Spatial connectivity and mechanics are described using an off-lattice, cell-centre model. Each cell is defined by position of its centre, enclosed by its Voronoi region. Cell-cell connectivity is determined through a Delaunay triangulation. Alternatively, a vertex-based model defines a cell as a collection of the vertices enclosing the cell area (in 2D). In Osborne et al. [37], a cell-centre and vertex-based model of the crypt is compared with a continuum description to investigate the propagation of mutations. More recently, in Osborne et al. [38] the cell-centre model and vertex-based model are studied in conjunction with other multicellular implementations: the cellular automata, as used in Loeffler et al. [39]; the Cellular Potts model, as considered in Osborne [40]; and the overlapping spheres model, which has been employed by Buske et al. [34]. In particular, for a case study concerning the colonic crypt, measurements for cell migration velocities are obtained for each implementation. These measurements will be used parametrise the subcellular models in later sections.

One notable aspect of the Van Leeuwen et al. study is the regulation of both proliferative capacity and cell adhesion by Wnt signalling. This two-fold influence is particularly important in the context of monoclonal conversion in the crypt; that is, the takeover of the crypt cell population by a single stem cell lineage, referred to as a clone. This phenomenon is implicated in the initiation of colorectal cancer, for any mutated cell must be able to persist long enough in the crypt to effect significant changes [41]. In Mirams et al. [42], an exhaustive computational study on the effect of proliferation and adhesion on monoclonal conversion in the crypt is performed. A telling result is that excessive proliferative capacity alone is insufficient for clonal dominance by a single mutant cell, and must coupled with an increase in cell adhesion in order to have a non-zero probability of clonal takeover occurring. This motivates the importance of being able to model both biochemical and biomechanical changes through subcellular pathway frameworks.

Van Leeuwen et al. [32] were the first to model the effect of Wnt signalling within a multicellular model of the crypt. The Wnt signalling pathway is perhaps the most important pathway present in the crypt, responsible for processes such as proliferation, adhesion, differentiation, and more [43]. It acts primarily to regulate the production and degradation of *β*-catenin within cells. Moreover, its disruption and hence misregulation of *beta*-catenin has been demonstrated to be crucial to colorectal cancer progression [44]. Consequently, there has significant mathematical modelling efforts to understand the Wnt pathway. A review of the features of key mathematical models, including the model used in Van Leeuwen et al. [30], can be found in Maclean et al. [45]. One model that we use in this study for its simplicity, which is not discussed in Maclean et al. that of Tan et al. [46]. In Tan et al., the authors derive a minimal model of the binding and shuttling of *β*-catenin between cellular compartments, using experimental data obtained by stimulating colon and kidney epithelial cells with Wnt and observing *β*-catenin concentration levels. We note that we do not consider more detailed subcellular models that model crosstalk between Wnt and other pathways, such as Notch, as in Kay et al. [47], and only focus on Wnt and its role in regulating *β*-catenin. Such models would form part of a future study.

### SBML

The Systems Biology Markup Language (SBML) [48] is the most commonly used standard for representing biochemical reactions, or gene-regulatory networks, and databases like BioModels [49] provide a huge variety of subcellular models for download. Specifically models for the Tan et al. [46] and Van Leeuwen et al. [30] are available in SBML (See Supplementary Files 1 and 2 respectively). SBML allows the specification of reactions independently of the method used to simulate them and allows differing simulation methods and tools to be compared.

### Overview

In this study we integrate subcellular reaction network models encoded in SBML, such as those of Van Leeuwen et al. [32] and Tan et al. [46], into multiscale models of crypts in the open source Chaste framework [30, 37]. By simulating these multiscale models, we can analyse how subcellular processes affect the mechanics and stability of the crypt as a whole. This is especially interesting when the subcellular processes in one cell (or a few single cells) undergo mutations which, through the multicellular coupling, enhance or inhibit cell growth, differentiation, and migration.

The remainder of this paper is structures as follows. In the “Results” section, we first present simple extensions to the subcellular Wnt pathway models of Van Leeuwen et al. [32] and Tan et al. [46] to include APC knockdown mutations. We then present the multiscale coupling used to develop a true multiscale model of the crypt. Finally this multiscale model is use to investigate the efficacy of cells with the mutant knockdown (referred to as mutant cells) to both invade and take over an otherwise healthy crypt. In the “Discussion” section we discuss how our results demonstrate how simple knockdowns in subcellular models can lead to invasive mutant cells and present avenues for future work. Full details of the multiple mathematical model components and the computational infrastructure used in this study (including the released open-source code) are given in the “Methods” section.

## Results

In this section we first present a multiscale model for a crypt, where subcellular reaction networks (represented in SBML) are incorporated into every crypt cell. We introduce mutation parameters and analyse the effect of mutations of cells on the mechanics of the crypt. Finally, we discuss the particular subcellular conditions necessary to generate persistence of mutant cells in the crypt.

### A multiscale model for mutant cells in the crypt

**Fig. 1:**
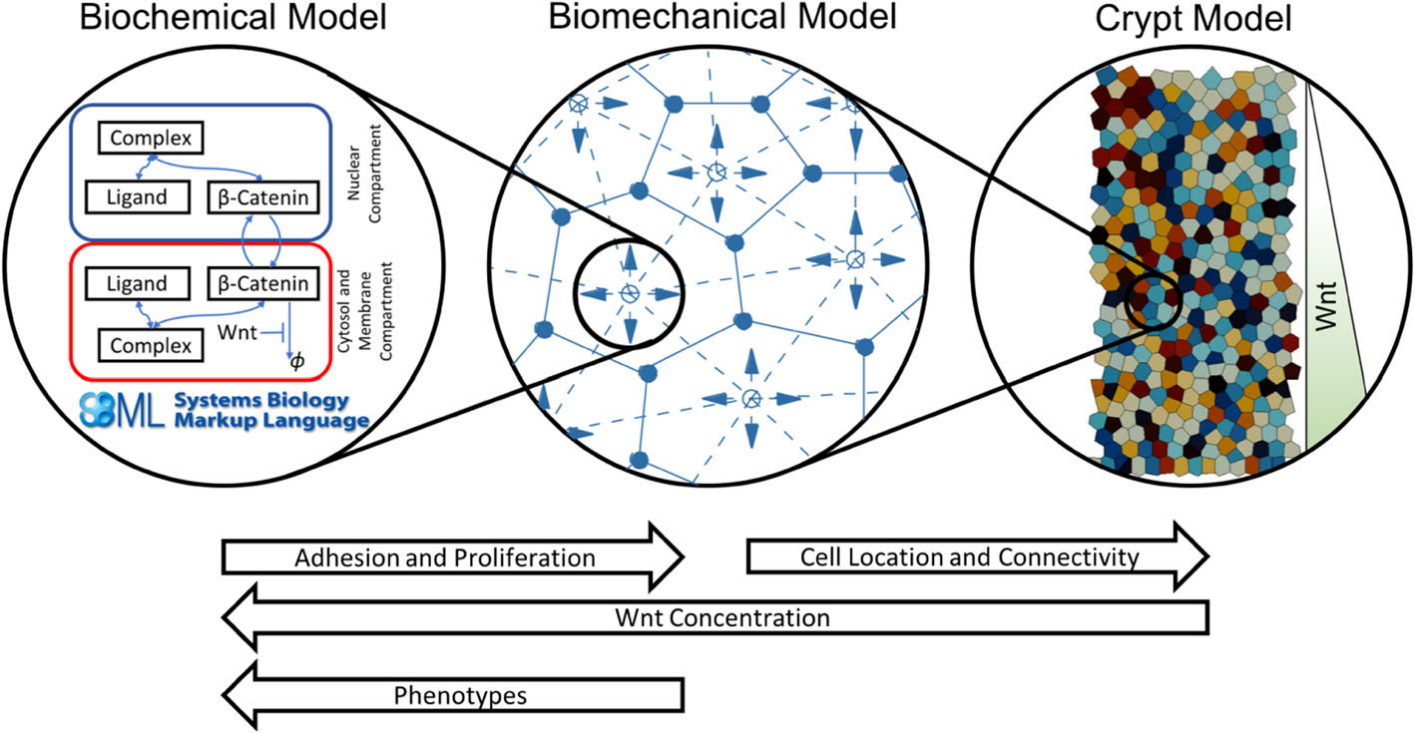
Schematic of the Model. The crypt model comprises a biomechanical model (the voronoi tessellation model found in Chaste) and a suitable biochemical model specified in SBML. The biochemical model specifies adhesion and cell proliferation in the biomechanical model which leads to cell movement in the crypt model. Cell phenotype (mutation level) and Wnt concentration are provided to the biochemical model by the biomechanical and crypt models respectively. SBML logo reproduced with permission from http://www.sbml.org

In order to analyse the effect of mutations in individual crypt cells to the biomechanics of a crypt as a whole, we developed a coupled multiscale model of a crypt, in a similar fashion to Van Leeuwen et al. [30] and Osborne et al. [37]. A schematic of model coupling is shown in Fig. 1 and is detailed below.

#### Crypt model

Following [37] we represent the crypt as a cylinder, 20 cell diameters (1 cell diameter is 10 *μ*m) long and 10 cell diameters in circumference, for convenience we represent this as an unfolded rectangular surface with periodic boundary conditions on the left and right. To represent the base of the crypt we impose a solid boundary there, and to represent sloughing into the lumen all cells that reach the top of the crypt are removed. specifically, once the vertical location of a cell reaches 20 cell diameters it is removed from the simulation. We impose a linear gradient of the signalling factor Wnt, which decreases linearly from 1 at the crypt base to 0 at the top. Cells are represented by their centres which are points free to move in the rectangular domain. Cell connectivity is defined by the delaunay triangulation of the cell centres. Cells move due to forces exerted by neighbouring cells and we consider motion to be over damped [50]. Therefore the equation of motion of each cell, **r**_*i*_, are given by [31],

1 $$ \eta_{i} \frac{\mathrm{d}\mathbf{r}_{i}}{\mathrm{d}t} = \mathbf{F}_{i}(t) = \sum_{j \in \mathcal{N}_{i}(t)} \mathbf{F}_{ij}(t),  $$

where the force between connected cells is given by

2 $$ \mathbf{F}_{ij}(t) = \mu_{ij} \hat{\mathbf{r}}_{ij}(t) \left(||\mathbf{r}_{ij}(t)|| - s_{ij}(t) \right),   $$

where *η*_*i*_, *μ*_*ij*_ are the drag coefficient of cell *i* and the spring constant between cells *i* and *j* respectively, **r**_*ij*_(*t*)=**r**_*j*_(*t*)−**r**_*i*_(*t*), and $\hat {\mathbf {r}}_{ij}(t)= \mathbf {r}_{ij}(t)/||\mathbf {r}_{ij}(t)||$.

We consider two types of cells, proliferative and non-proliferative (or differentiated). Following [37], proliferative cells have a cell cycle duration draw from a Truncated Normal *N*(12,1) distribution comprised of the following phases: M phase (1 h); G1 phase (*N*(2,1) truncated so positive); S phase (5 h); and G2 phase (4 h). On completion of the cell cycle the cell divides creating two daughter cells which are placed at a distance of 0.1 cell diameters apart, about the parent cell, in a randomly chosen direction. We note that the resting spring length between the two daughter cells increases linearly with time until the cells are mature (end of M phase), so as to allow proper relaxation of the tissue.

#### Model of the wnt pathway

**Fig. 2:**
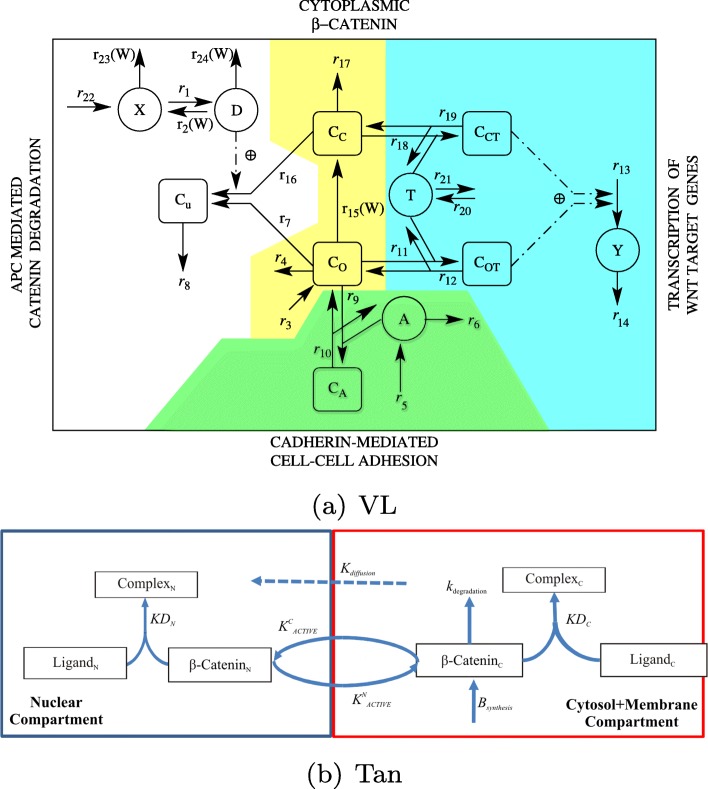
Subcellular model schematics. a) Schematic representation of the VL model, which describes the dynamics of active destruction complexes (*D*), axin molecules (*X*), adhesion molecules (*A*), transcription molecules (*T*), Wnt target proteins (*Y*) and six pools of *β*-catenin (*C*_*i*_, *i*=*A*,*c*,*c*_*T*_,*o*,*o*_*T*_ and *u*). Reproduced with permission from [32]. b) Schematic representation of the Tan model which describes the dynamics of free and bound *β*-catenin in the cytosol-membrane (*B*_*C*_, *N*_*C*_) and nucleus (*B*_*N*_, *N*_*N*_), and free ligands in the cytosol-membrane (*L*_*C*_) and nucleus (*L*_*N*_). Reproduced with permission from [46]

In recent years various models of subcellular reactions (in particular the canonical Wnt Pathway) in the crypt cells have been presented [45]. In this study we select two models. The model developed by Van Leeuwen et al. in 2007 [32] (referred to as the VL model), and the model presented by Tan et al. in 2014 [46] (referred to as the Tan model). Both models consider the degradation of *β*-catenin in the absence of Wnt and its dual role in cadherin-mediated cell adhesion and nuclear transcription. Loosely speaking, in both models, high concentrations of Wnt lead to high levels of nuclear *β*-catenin (i.e. *N*_*N*_ in the Tan model and *C*_*T*_ in the VL model) and cells progress through the cell cycle. Conversely, in both models, under low concentrations of Wnt cells differentiate. In each model we introduce a mutation parameters *γ*, which causes a knockdown to an appropriate reaction in each mode to represent APC disfunction (note *γ*=1 represents healthy APC and *γ*<1 represents disfunction). Both models are illustrated in Fig. 2 and full model details are given in the “Methods” section.

#### Multiscale coupling

To describe the effect of transcription complexes on cell division, in a similar way to [30], we incorporate a division threshold into the multiscale model, called the complex proliferative threshold. At each time step during a simulation, we check whether the amount of transcription complexes is above the complex proliferative threshold for every cell (i.e. *N*_*N*_ in the Tan model and *C*_*T*_ in the VL model, see “Methods” section for full details). If that is the case, the cell is classified as a proliferative cell which is able to move through the cell cycle. If it is not, the cell is becomes and stays differentiated for the remainder of the simulation. For both models, we choose the complex proliferative threshold such that in steady state (not mutated), the maximum height of the proliferative cells is approximately a quarter of the total crypt height. In the “Methods” section (Fig. 8), we can see that mutations give rise to more transcription complexes, which makes it easier to pass the complex proliferative threshold, leading to an increase in proliferation in the crypt. We chose proliferative thresholds so that, at steady state, healthy (*γ*=1) cells with a Wnt-level higher than 0.75 will proliferate, this corresponds to thresholds of *C*_*T*_=11.667 and *N*_*N*_=739.949 for the VL and Tan models respectively.

Since the number of bound adhesion complexes affects the ability of a cell to attach itself to neighbouring cells, we describe the drag experienced by each cell in the crypt as a function of the number of bound adhesion complexes. We calculate the drag of each cell to be between 1 and 20, and we require that if the cell is not mutated (i.e. *γ*=1), the drag of the cell is 1.

For both models, we define the drag of each cell *i* to be

3 $$ \eta_{i} = \text{max} \left(\left\{ \frac{A_{\mathrm{X}} - \alpha_{\mathrm{X}}}{\beta_{\mathrm{X}}}, 1 \right\} \right),   $$

where *α*_X_ and *β*_X_ are parameters to control the effect of adhesion complex, *A*_X_, on drag for each model, for X=VL and Tan. For the VL model the adhesion complex is given by *A*_VL_=*C*_*A*_ and for the Tan model *A*_Tan_=*N*_*C*_. For each model we fit *α*_X_ and *β*_X_ so that *η*_*i*_=1 for (*W*,*γ*)=(1,1) and *η*_*i*_=20 for (*W*,*γ*)=(1,0), (for the subcellular model at equilibrium) these parameters are given in Table 1.

**Table 1:** Drag Function Parameter Values

*α*_Tan_	697.82302
*β*_Tan_	14.45674
*α*_VL_	98.84798
*β*_VL_	3.98617

**Fig. 3:**
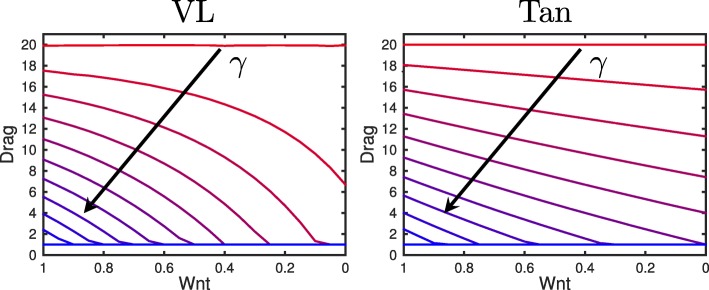
Drag functions. Drag as a function of the level of complex in the cytosol/membrane (*C*_*A*_ in the VL model and *N*_*C*_ in the Tan model). The lower *γ*, the higher the drag. I.e. for mutant cells the amount of complex in the cytosol/membrane increases, as seen in Fig. 8, and we assume that this increases the drag. Also when the Wnt signal is higher, the drag will be higher for *γ*≠0 (as the amount of complex is higher). Arrows are drawn in the direction of increasing *γ*. The cases are shown for *γ*=0−1 in increments of 0.1 (*γ*=0 is red, *γ*=1 is blue)

These drag functions are plotted in Fig. 3 for varying *γ*. We can see that for both models, when the mutation parameter is lower, the drag is higher, and when Wnt is lower, the drag is lower.

### Mutations cause overcrowding in homogeneous crypts by enlarging the proliferative compartment and increasing adhesion.

**Fig. 4:**
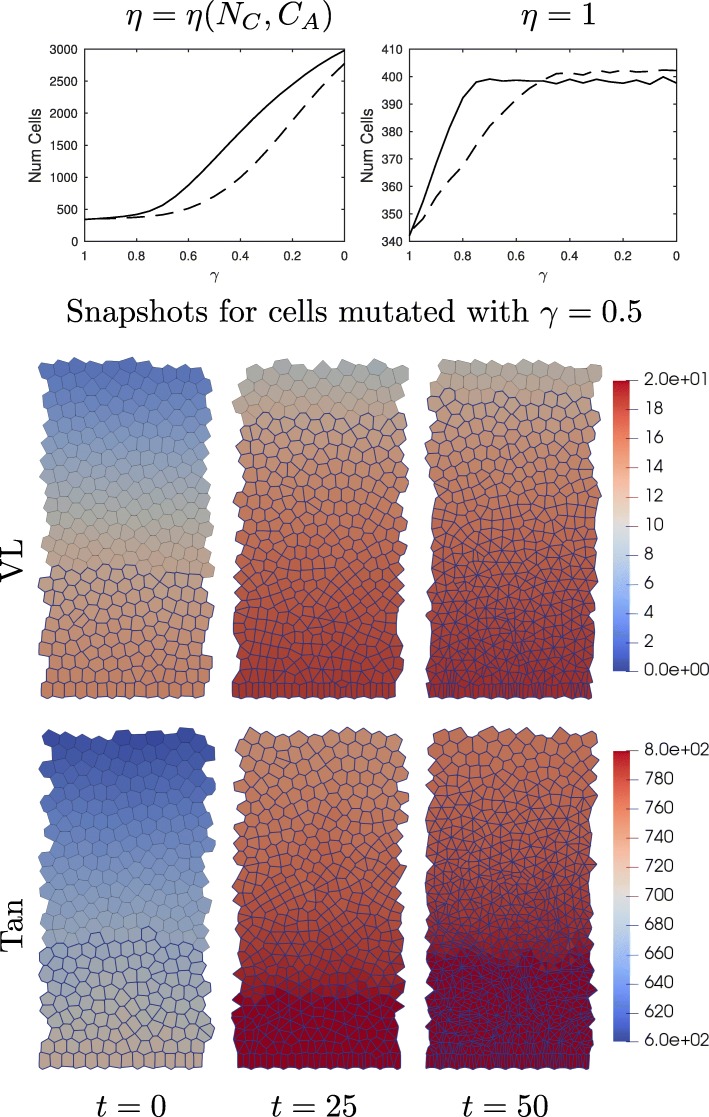
Number of cells in the crypt as a function of mutation parameter. Top) Averaged over 100 simulations for constant (*η*=1) and variable drag (Equation (3)). All cells are mutated in a dynamic equilibrium, at time *t*=0, the number of cells are counted at *t*=50 hours. Dashed lines are with the VL model and solid line are with the Tan model. Bottom) Snapshots of simulations using the adhesion complex dependent drag functions defined in Equation (3). (random seed = 0). Proliferative cells are outlined in blue and cell color indicates level of nuclear complex. All cells in the crypts are mutated at time *t*=0h, with parameter *γ*=0.5, for both models

In order to see how changing *γ* influences the structure of the crypts we run 100 sets of simulations with VL and Tan subcellular reaction networks using both a uniform drag (all cells have a drag *η*=1) and a adhesion complex dependent drag as given in Eq. (3). Initially we run the model until the crypt has reached dynamic equilibrium (the subcellular models have reached a steady state and the number of proliferating cells remains approximately the same), here *t*=500h. Then we mutate all cells uniformly by changing the parameter *γ*. Once all cells have been mutated we track the number of cells in the crypt for a further 50 h. The averaged results outlining the number of cells in the crypt are presented in Fig. 4.

For both uniform drag of *η*=1 and the new drag function, *η*=*η*(*N*_*C*_,*C*_*A*_), the number of cells increases more rapidly in the Tan model (solid line) than in the VL model (dashed line) as *γ* decreases (i.e. the level of mutation increases), and the proliferative height reaches full crypt height more quickly (which means the crypt is completely populated with proliferative cells). Since our new drag function, *η*=*η*(*N*_*C*_,*C*_*A*_), can increase the drag of cells in the crypt based on a cell’s expression of adhesion complexes, the number of cells in the crypt increases drastically for these simulations. As the number of cells increases the average size of a cell is reduced, this is in line with homogeneous human derived tissue cultures as seen in [51] where cells with an APC mutation in isolation are approximately 25% smaller than their non mutated counterparts.

In Figure 4 a snapshots of single simulations are included for each model, and we can see that the crypts become overpopulated with proliferative cells due to the increased drag caused by increased complex in the cytosol/membrane. This is due to: i) the proliferative compartment enlarging and ii) the drag coefficient increasing.

### Mutations allow cells to persist in the crypt.

**Fig. 5:**
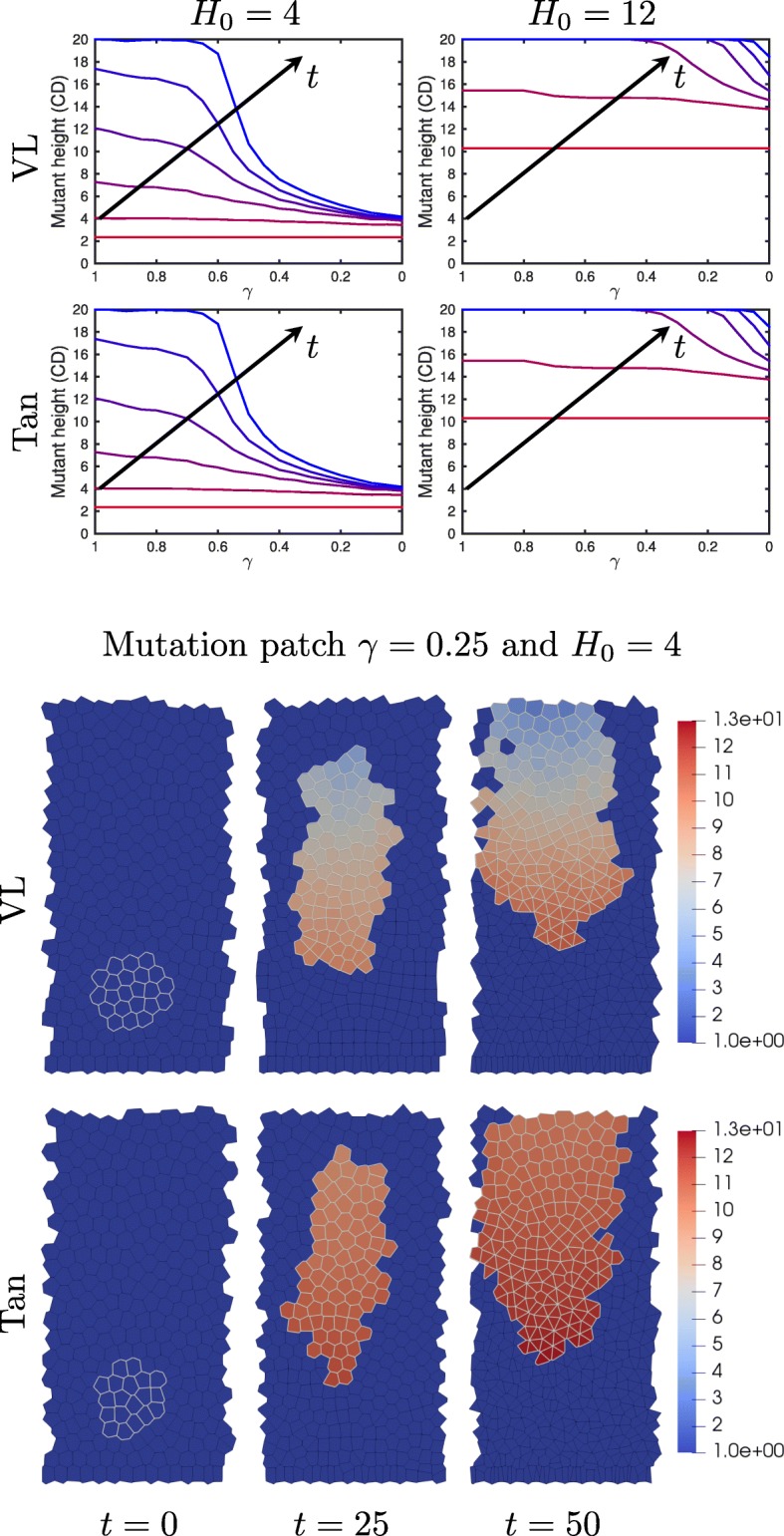
Mutant cells remain in the crypt due to increased adhesion and proliferation. Top) Mutant height plotted at times *t*=0, 10, 20, 30, 40, 50h after mutating cell. The simulations start with a mutant patch of radius 2, and initial centre height *H*_0_. The mutant height is given in cell diameters. Bottom) Snapshots of simulations with mutant cells at bottom of crypt, *H*_0_=4. (random seed = 0). Mutated cells are outlined in grey and the cell color indicates level of drag coefficient, *η*, applied to the cell (function of adhesion complex). Mutant cells in the crypts are mutated at time *t*=0h, with parameter *γ*=0.25, for both models

To analyse the effect, on the crypt, of mutations in a few single cells, we start with a crypt in steady state and mutate patches of cells, in analogue to [37]. The centre of these mutant patches is given by *H*_0_ and we mutate all cells within a radius of 2 cell diameters (20 *μ*m) and track their positions over time. Figure 5 top) shows the minimum height of mutant cells in the crypt at varying times after mutation. We see that as *γ* decreases, the minimum mutant height decreases and the mutations are more persistent. As the initial mutation patch height increases to *H*_0_=12 cell diameters, the mutations become less persistent for low levels of mutation (*γ*>0.3) and the minimum mutation height increases faster with mutated cells being sloughed off the crypt during the simulation. However, we still see cells persisting in the crypt for severe APC disfunction (*γ*≈0).

In the bottom of Fig. 5, a few snapshots are shown of simulations with *γ*=0.25, and we see that in both models the mutant patch grows and moves upwards but is decelerating. However, since *γ* has a stronger effect in the Tan model, we see that the mutant patch has grown more in the crypt with cells of the Tan model. This corresponds to the results of the number of transcription complexes in a single cell as shown before in Fig. 8. For *γ*=0.25, the levels of adhesion and transcription complexes in the cell are relatively higher in the Tan model at lower Wnt levels than for *γ*=0.25 in the VL model, hence generating more adhesion and more transcription higher up the crypt.

### Coupling mechanical properties to adhesion complex allows mutant cells to invade the crypt.

In order to investigate how expression of the APC mutation parameter, *γ*, influences the invasive properties of cells, we consider how effective mutant cells are in taking over the crypt. We start with a crypt at dynamic equilibrium (*t*=500h) and mutate a randomly selected fraction of cells. Subsequently we track cell numbers in the crypt until all cells in the crypt are either healthy or mutant (or the mutant cells have caused a large increase in the number of cells in the crypt, as seen in Fig. 4, which we take to mean that mutant cells have taken over the crypt). Repeating this multiple times for each value of *γ*, and varying the initial percentage of mutant cells, allows us to calculate the probability of mutant cells (with a specific *γ*) taking over the crypt (similar investigations are undertaken, on different crypt models, in [36]).

**Fig. 6:**
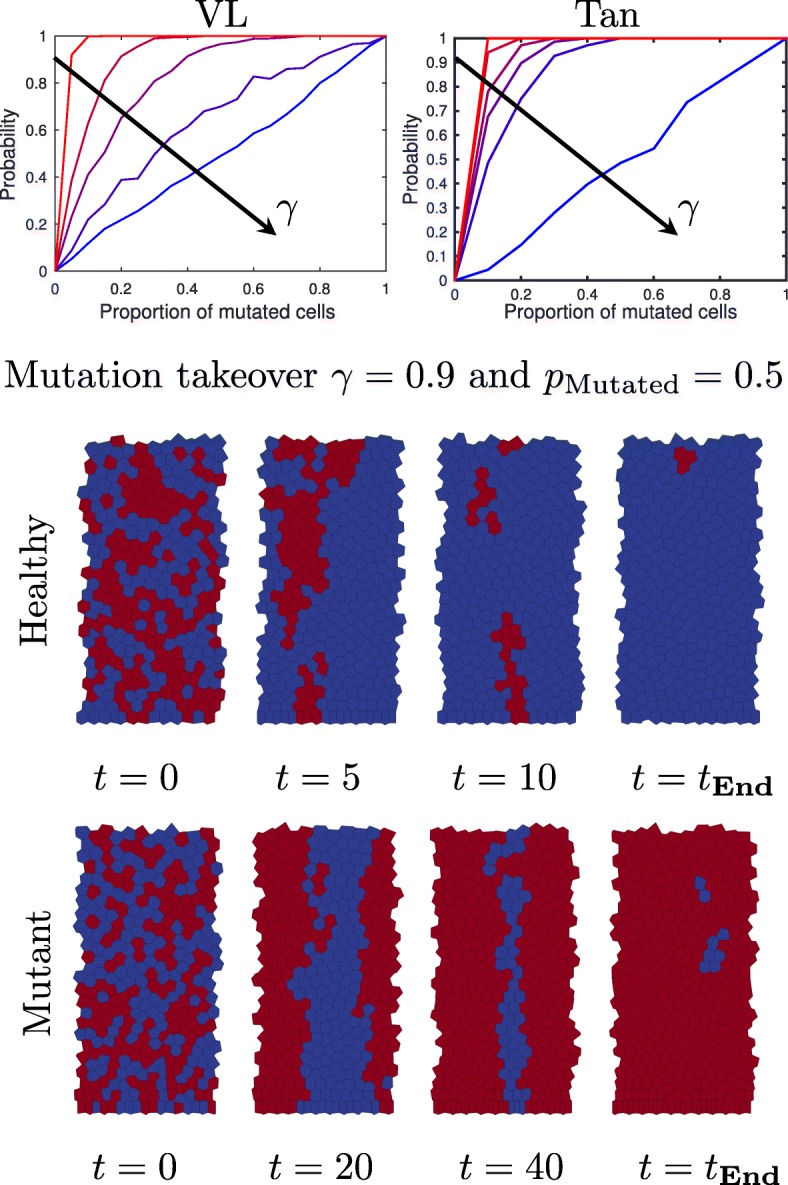
Mutant cells invade the crypt. Top) probability of mutant cells taking over the crypt as a function of initial proportion of mutated cells, for the VL and Tan models as the mutation parameter *γ* is varied. results are presented for *γ*=0 (red),0.5,0.75,0.9, and 1 (blue) and the arrow is in the direction of increasing *γ* Bottom) Snapshots of 2 simulations with 50% mutated cells. In the top simulation healthy cells happen to out compete the mutant cells, in the bottom simulation mutant cells win. Note, specific simulations were chosen to illustrate the conversion to homogeneous crypts

Figure 6 shows how the probability of invasion varies as we change the expression of the APC mutation parameter, *γ*. In both models we see that as *γ* is decreased (i.e. the level of mutation is increased) the mutant cells become more effective at invading the crypt. In order to see what influence the different components of the model have on the invasive ability, we rerun this mutant takeover study for cells with homogeneous mechanical properties (i.e *η*=1) in this case we see that there is no advantage from mutation (results not shown here for brevity). Therefore, when cells have identical mechanical properties mutated cells no longer have an invasive advantage.

Snapshots for simulations where healthy or mutant cells takeover the crypt are shown in the bottom of Fig. 6. For the chosen parameters (*γ*=0.9) a crypt initially comprising an equal number of mutant and healthy cells is twice as likely to be taken over by mutant cells than healthy cells. If we used homogeneous drag *η*=1 then it would be equally likely to be taken over by either healthy or mutant cells, which is in agreement with Mirams et al. [42].

**Fig. 7:**
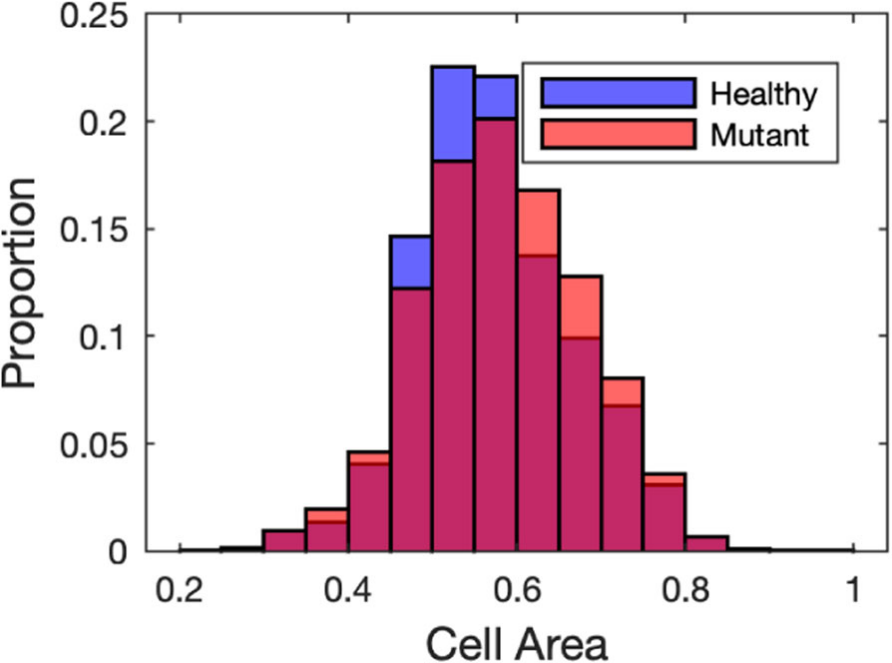
Mutant cells maintain cell volume in homogeneous crypts. Distribution of healthy and mutant cell areas for heterogeneous crypts from Fig. 6 using the Tan model for cell signalling. Cell age is measured after 20 hours within 100 crypts initially comprised of an equal quantity of healthy (*γ*=1) and mutated cells (*γ*=0.5). The mean healthy cell area is 0.5725 CD^2^, and the mean mutant cell area is 0.5826 CD^2^

In order to see the effect that mutation has on the mechanics (in particular deformation) of cells we track the area of each cell type (healthy *γ*=1, and mutant *γ*≠1) in the heterogeneous crypts from the simulations in Fig. 6. In Figure 7 we present histograms showing the distribution of cell sizes of healthy (*γ*=1) and mutant (*γ*=0) cells after 20 h in a crypt (with Tan Model) initially comprised of equal numbers of healthy and mutant cells (these are the same simulations as in Fig. 6). We see that in heterogeneous crypts mutant cells are larger than healthy cells. This is in contrast to when we have homogeneous crypts, where cells with higher levels of mutation (i.e lower *γ*) are smaller (see Fig 4). These larger mutant cells are the ones that will persist in the crypt as shown in Fig. 6.

**Fig. 8:**
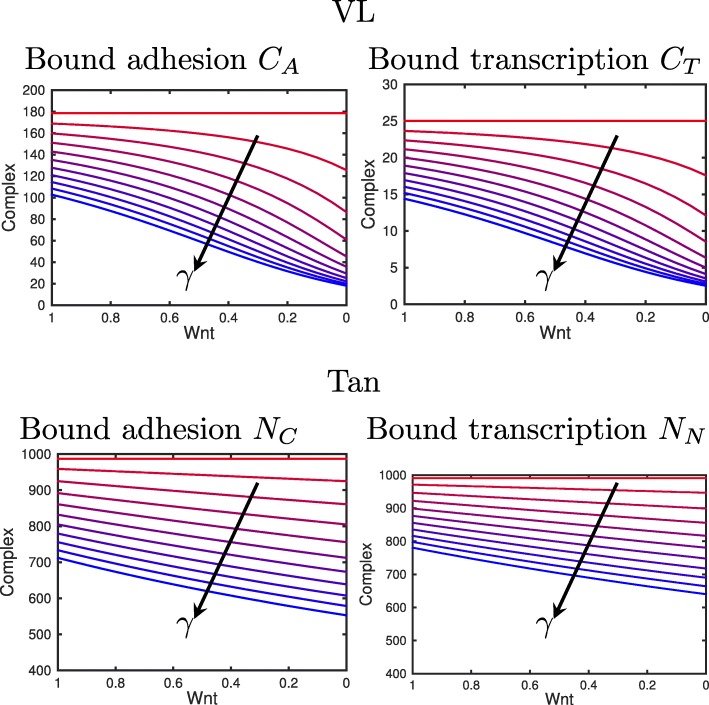
The effect of varying the expression of the APC mutation parameter, *γ*, on the bound complexes in the nucleus and cytosol-membrane. For the VL (top) and Tan models (bottom). Arrows are drawn in the direction of increasing *γ*. Decreasing *γ* increases the amount of bound complexes in both the complexes for adhesion and transcription. The cases are shown for *γ*=0−1 in increments of 0.1 (*γ*=0 is red, *γ*=1 is blue)

## Discussion

In this paper we have presented a framework which enables arbitrary subcellular models to be incorporated into a multicellular model for crypt dynamics. The framework has been used to study how mutations in subcellular dynamics manifest at the tissue level. In particular we have shown how mutations which increase the number of adhesion complexes increase both the persistence and invasion power of cells.

We saw that the VL and Tan models exhibit similar behaviour for all results presented in this paper. This is due to the fact that while different, both models are representing the same pathway and mechanism of mutation. This study could be extended to attempt to equate parameters between the models, which would be especially of interest as the parameters in the Tan model are fitted to data [46].

In this study, we have modelled both proliferation and adhesion to depend on Wnt signalling through a subcellular pathway specified by SBML. However, we have not modelled any feedback mechanisms between the proliferative ability and mechanical properties of cells. This framework may readily be extended to incorporate mechanical regulation of proliferation and other cellular behaviours through other subcellular models. One such pathway that could be considered is the YAP/TAZ pathway, a mechanotransductive pathway that has been to demonstrated to affect *β*-catenin nucleation within the crypt, in turn affecting the proliferation [52, 53]. This would clearly have an influence on the emergent effects of the Wnt pathways considered in this paper. As the notion that cellular behaviours such as proliferation and differentiation are also regulated by mechanical forces through mechanotransduction and vice versa [54, 55], multicellular models will need to be able to incorporate such processes readily and efficiently.

The approach to modelling the influence of mutations on cell interactions used here is the one established in Van Leuweenn et al. [30] and Osborne et al. [37]. This method assumes that cell attraction and repulsion is affected by mutation in the same way. Cell repulsion is mainly used to represent the physical exclusion of two contracting cells whereas cell attraction represents the interaction of surface bound adhesion molecules. Therefore, it may be appropriate to consider the effect of mutation on attraction and repulsion separately and this will form the basis of a future investigation.

## Conclusions

The framework presented in this paper allows any model for the Wnt pathway that is specified in SBML (or can be specified in SBML) to be coupled to a multicellular model of the crypt. Using the framework we can see how subcellular mutations i.e. knockouts or knockdowns, affect cell level properties and the resultant migration of cells within epithelial tissues. Specifically, we are able to specify rate parameters in the subcellular model of individual cells (or groups of cells) and through the multiscale coupling we see how these mutations affect the movement of cells. This allows us to show that mutations in APC can bias neutral drift and cause downward invasion of mutant cells in the crypt.

Crucially, the framework is freely available under an open source licence (see “Methods” section). Therefore, using the tools developed here researchers will be able to implement arbitrary subcellular reaction networks in multicellular systems enabling more realistic simulations to be undertaken. While used here to investigate the efficacy of mutations in the colorectal crypt the tools developed here can be used for other biological systems.

## Methods

The computational models for the subcellular reactions in the crypt cells used in this study are the model developed by Van Leeuwen et al. in 2007 [32], and the model presented by Tan et al. in 2014 [46].

### The Van leeuwen (2007) model of crypt cells

In this section, we outline the original dimensional one-compartment model of *β*−catenin regulation presented by Van Leeuwen et al. (2007). It describes the dynamics of six forms of *β*-catenin *C*_*i*_,*i*=*A*,*c*,*c*_*T*_,*o*,*o*_*T*_,*u*, destruction complexes *D*, axin *X*, transcription molecules *T*, adhesion molecules *A*, and Wnt target proteins *Y*. The open form of *β*-catenin is described by *C*_*o*_, which can undergo APC-mediated degradation by the destruction complex and APC-independent degradation, bind adhesion molecules, undergo APC-mediated phosphorylation, and form a closed form of *β*-catenin *C*_*c*_. The APC-mediated degradation of *C*_*o*_ and *C*_*c*_ first produces *C*_*u*_ by phosphorylation, which is subsequently marked for ubiquitination. Both *C*_*o*_ and *C*_*c*_ can bind to transcription molecules to produce $C_{o_{T}}$ and $C_{c_{T}}$, respectively. *C*_*c*_ is also degraded independently of APC. Bound *β*-catenin to adhesion complexes (i.e. complexes of *C*_*o*_ and *A*) are described by *C*_*A*_, which is also subject to APC-independent degradation.

APC-mediated degradation of *β*-catenin:

4 $$\begin{array}{@{}rcl@{}} \frac{d D}{d t} &=& \gamma s_{D} X - \left(\hat{d}_{D}(W) + \hat{d}_{D x}(W)\right) D,  \end{array} $$

5 $$\begin{array}{@{}rcl@{}} \frac{d X}{d t} &=& s_{X} - \gamma s_{D} X - \hat{d}_{X}(W) X  \\ && + \hat{d}_{Dx}(W) D,  \end{array} $$

6 $$\begin{array}{@{}rcl@{}} \frac{d C_{u}}{d t} &=& \frac{ p_{u} D C_{F}}{C_{F} + K_{D}} - d_{u} C_{u},  \end{array} $$

Cytoplasmic *β*-catenin:

7 $$\begin{array}{@{}rcl@{}} \frac{d C_{o}}{d t} &=& s_{C} + d_{CA}C_{A} + d_{CT} C_{o_{T}}  \\ && - \left(s_{CA} A + s_{CT} T + d_{C}\right) C_{o}  \\&& - \frac{\hat{p}_{c}(W) C_{o}}{C_{o} + K_{c}} - \frac{ p_{u} D C_{c}}{C_{F} + K_{D}},  \end{array} $$

8 $$\begin{array}{@{}rcl@{}} \frac{d C_{c}}{d t} &=& \frac{\hat{p}_{c}(W) C_{o}}{C_{o} + K_{c}} + d_{CT} C_{c_{T}}  \\ && - \left(s_{CT} T + d_{C}\right) C_{c} - \frac{p_{u} D C_{c}}{C_{F} + K_{D}},  \end{array} $$

E-cadherin-mediated cell-cell adhesion:

9 $$\begin{array}{@{}rcl@{}} \frac{d A}{d t} &=& s_{A} + d_{CA} C_{A} - \left(s_{CA} C_{o} + d_{A}\right) A, \end{array} $$

10 $$\begin{array}{@{}rcl@{}} \frac{d C_{A}}{d t} &=& s_{CA} C_{o} A - d_{CA} C_{A}, \end{array} $$

Transcription of Wnt-target genes:

11 $$\begin{array}{@{}rcl@{}} \frac{d T}{d t} &=& s_{T} + d_{CT} C_{T} - (s_{CT} C_{F} + d_{T}) T, \end{array} $$

12 $$\begin{array}{@{}rcl@{}} \frac{d C_{o_{T}}}{d t} &=& s_{CT} C_{o} T - d_{CT} C_{o_{T}}, \end{array} $$

13 $$\begin{array}{@{}rcl@{}} \frac{d C_{c_{T}}}{d t} &=& s_{CT} C_{c} T - d_{CT} C_{c_{T}}, \end{array} $$

14 $$\begin{array}{@{}rcl@{}} \frac{d Y}{d t} &=& \frac{s_{Y} C_{T}}{C_{T} + K_{T}} - d_{Y} Y,  \end{array} $$

In the above equations, $\phantom {\dot {i}\!}C_{F} = C_{o} + C_{c}$ and $C_{T} = C_{o_{T}} + C_{c_{T}}\phantom {\dot {i}\!}$. The synthesis rates of the substrates/complexes are described by the symbols *s*_*i*_, with substrate/complexes as subscript. The degradation rates are described by *d*_*i*_ and $\hat {d}_{i}$. The symbol $\hat {p}_{c}$ represents the phosphorylation rate of *C*_*o*_ to its closed form *C*_*c*_. The symbol *p*_*u*_ describes the rate of phosphorylation of *C*_*o*_ and *C*_*c*_ by the destruction complex. Note that there are various saturation coefficients in the model, i.e. *K*_*D*_, *K*_*c*_ and *K*_*T*_. The phosphorylation rates of *C*_*o*_ and *C*_*c*_ by the destruction complex has Michaelis-Menten saturation coefficient *K*_*D*_. The rate of production of Wnt target proteins *Y* has Michaelis-Menten saturation coefficient *K*_*T*_. Lastly, *K*_*c*_ is the saturation constant for the rate of production of *C*_*c*_ from *C*_*o*_ by phosphorylation.

The Wnt signal, denoted by 0≤*W*≤1, is incorporated into the model in four parameters which are indicated in the model equations by hat symbols. It is expressed in $\hat {d}_{Dx}(W) = d_{Dx} + \xi _{Dx} W$ (dissociation of destruction complexes), $\hat {d}_{D}(W) = d_{D} + \xi _{D} W$ (elimination of active destruction complexes by mechanisms other than dissociation), and $\hat {d}_{X}(W = d_{X} + \xi _{X} W$ (elimination/destabilisation of free axin). Van Leeuwen et al. [32] consider two hypotheses. Under the first hypothesis, we have that $\hat {p}_{c}(W) = p_{c}$ and Wnt does not influence the formation of closed-form *β*-catenin *C*_*c*_ from open-form *β*-catenin *C*_*o*_. Under the second hypothesis, Wnt enhances the production of closed-form *β*-catenin *C*_*c*_ by increasing the rate of phosphorylation which converts *C*_*o*_ to *C*_*c*_. This is expressed by $\hat {p}_{c}(W) = p_{c} + \xi _{c} W$. For the purposes of this paper, we selected and worked with the first hypothesis, i.e. $\hat {p}_{c}(W) = p_{c}$ but either could be used.

Following [32], we have introduced a mutation parameters *γ*. This mutation represents APC disfunction, by multiplying *s*_*D*_ by 0≤*γ*<1 in Eqs. (4) and (5). This leads to a reduction in the rate at which new destruction complexes are formed. Van Leeuwen et al. [32] denote this mutation as ‘apc1’, but only consider a 50% and 100% reduction in APC function (i.e. *γ*=0.5 and *γ*=0, respectively).

The authors also introduce a second mutation which reflects a mutation in the recognition of *β*-catenin by *GSK*3*β*, by multiplying *p*_*u*_ by 0≤*γ*<1 in Eqs. (6)–(8). This reduces *β*-catenin phosphorylation by the destruction complex. Van Leeuwen et al. [32] denote this mutation as ‘mut2’, but only consider a 100% reduction in phosphorylation. The effect of this second mutation is very similar to the mutation considered here so we restrict ourselves to the first mutation.

We denote the set of steady states by $\left \{\widetilde {D}, \widetilde {X}, \widetilde {C}_{u}, \widetilde {C}_{o}, \widetilde {C}_{c}, \widetilde {A}, \widetilde {C}_{A}, \widetilde {T}, \widetilde {C}_{o_{T}}, \widetilde {C}_{c_{T}}, \widetilde {Y}\right \}$. The steady states of the Van Leeuwen system of ODEs are

15 $$\begin{array}{@{}rcl@{}} \widetilde{D} &=& \frac{\gamma_{1} s_{D} s_{X}}{\gamma s_{D} \hat{d}_{D} + \hat{d}_{X} \left(\hat{d}_{D} + \hat{d}_{Dx}\right)},  \end{array} $$

16 $$\begin{array}{@{}rcl@{}} \widetilde{X} &=& \frac{\widetilde{D} \left(\hat{d}_{D} + \hat{d}_{dx}\right)}{\gamma s_{D} }, \end{array} $$

17 $$\begin{array}{@{}rcl@{}} \widetilde{C}_{u} &=& \frac{p_{u} \widetilde{D} \widetilde{C}_{F}}{d_{u} \left(\widetilde{C}_{F} + K_{D}\right)}, \end{array} $$

18 $$\begin{array}{@{}rcl@{}} \widetilde{C}_{o} &=& \frac{s_{C}-\hat{p}_{c} - z^{*} K_{C}}{2 z^{*}} \\ && + \frac{\sqrt{s_{C}^{2} \left(1-\frac{\hat{p}_{c}}{s_{C}} - \frac{z^{*} K_{C}}{s_{C}}\right)^{2} + 4 s_{C} z^{*} K_{C}}}{2 z^{*}}, \end{array} $$

19 $$\begin{array}{@{}rcl@{}} \widetilde{C}_{c} &=& \widetilde{C}_{F} - \widetilde{C}_{o}, \end{array} $$

20 $$\begin{array}{@{}rcl@{}} \widetilde{A} &=& s_{A} / d_{A}, \end{array} $$

21 $$\begin{array}{@{}rcl@{}} \widetilde{C}_{A} &=& \widetilde{A} s_{CA} \widetilde{C}_{o} / (d_{CA}), \end{array} $$

22 $$\begin{array}{@{}rcl@{}} \widetilde{T} &=& s_{T} / d_{T}, \end{array} $$

23 $$\begin{array}{@{}rcl@{}} \widetilde{C}_{o_{T}} &=& s_{CT} \widetilde{T}\widetilde{C}_{o} / (d_{CT}), \end{array} $$

24 $$\begin{array}{@{}rcl@{}} \widetilde{C}_{c_{T}} &=& \widetilde{C}_{o_{T}}\widetilde{C}_{c} / \widetilde{C}_{o}, \end{array} $$

25 $$\begin{array}{@{}rcl@{}} \widetilde{Y} &=& \frac{s_{Y} s_{T} s_{CT} \widetilde{C}_{F}}{d_{Y} \left(s_{T} s_{CT} \widetilde{C}_{F} + d_{T} d_{CT} K_{T}\right)}, \end{array} $$

with $\widetilde {C}_{F} = \frac {s_{C} - d_{C} K_{D} - p_{u} \widetilde {D}}{2 d_{C}} + \frac {\sqrt {s_{C}^{2} \left (1 - \frac {d_{C} K_{D}}{s_{C}} - \frac {p_{u} \widetilde {D}}{s_{C}}\right)^{2} + 4 s_{C} d_{C} K_{D}})}{2 d_{C}}$ and $z^{*} = d_{C} + p_{u} \widetilde {D} / \left (\widetilde {C}_{F} + K_{D}\right)$.

### The tan (2014) model of crypt cells

The two-compartment model of *β*−catenin regulation presented by Tan et al. (2014) considers the dynamics of free and bound *β*-catenin in the cytosol-membrane and nucleus, and free ligands in the cytosol-membrane and nucleus. Free *β*-catenin is denoted by *B* and superscripted by either *C* (cytosol-membrane) or *N* (nucleus). Likewise, bound *β*-catenin is denoted by *N* and superscripted by either *C* or *N*. The free ligands in the nucleus denoted by *L*_*N*_ only consist of transcription molecules, while the free ligands in the cytosol-membrane denoted by *L*_*C*_ only represent adhesion molecules.

The model equations are as follows:

26 $$\begin{array}{@{}rcl@{}} \frac{dB_{C}}{dt} &=& B_{\text{synth}} - {k_{\text{deg}}\gamma\left(1-\frac{W}{2}\right) B_{C}}  \\&&+ k_{R}^{C}C_{C} - k_{F}^{C}B_{C}L_{C} - \frac{k_{\text{diff}}(B_{C}-B_{N})}{V_{C}}  \\ && -\frac{k_{\text{active}}^{C}B_{C}-k^{N}_{\text{active}}B_{N}}{V_{C}},  \end{array} $$

27 $$\begin{array}{@{}rcl@{}} \frac{dB_{N}}{dt} &=& k_{R}^{N} N_{N} - k_{F}^{N} B^{N} L_{N}  \\&&+ \frac{k_{\text{diffusion}}(B_{C}-B_{N})}{V_{N}}  \\&&+ \frac{k^{C}_{\text{active}}B_{C}-k^{N}_{\text{active}}B_{N}}{V_{N}}, \end{array} $$

28 $$\begin{array}{@{}rcl@{}} \frac{dL_{C}}{dt} &=& k_{R}^{C} N_{C}-k_{F}^{C} B_{C} L_{C}, \end{array} $$

29 $$\begin{array}{@{}rcl@{}} \frac{dN_{C}}{dt} &=& -k_{R}^{C} N_{C} + k_{F}^{C} B_{C} L_{C}, \end{array} $$

30 $$\begin{array}{@{}rcl@{}} \frac{dL_{N}}{dt} &=& k_{R}^{N} N_{N} - k_{F}^{N} B_{N} L_{N}, \end{array} $$

31 $$\begin{array}{@{}rcl@{}} \frac{dN_{N}}{dt} &=& -k_{R}^{N} N_{N} + k_{F}^{N} B_{N} L_{N}.  \end{array} $$

As in our simulations with the Van Leeuwen model, *W* is the level of Wnt and we have introduced a parameter 0≤*γ*<1 in Eq. (26) to simulate the effect of mutations to a cell. Eqs. (28)– (31) lead to the following conditions

32 $$\begin{array}{@{}rcl@{}} L_{C} + N_{C} &=& L_{C_{T}},  \end{array} $$

33 $$\begin{array}{@{}rcl@{}} L_{N} + N_{N} &=& L_{N_{T}},  \end{array} $$

where $L_{C_{T}}$ and $L_{N_{T}}$ are known constants independent of time. We denote the set of steady states by $\left \{\widetilde {B}_{C}, \widetilde {B}_{N}, \widetilde {L}_{C}, \widetilde {L}_{N}, \widetilde {N}_{C}\, \widetilde {N}_{N}\right \}$. Solving Eqs. (28) and (32) simultaneously yields

34 $$\begin{array}{@{}rcl@{}} \widetilde{L}_{C} = \frac{L_{C_{T}} k_{R}^{C}}{k_{R}^{C}+k_{F}^{C}\widetilde{B}_{C}}\text{,} \quad \widetilde{N}_{C} = \frac{L_{C_{T}} k_{F}^{C}\widetilde{B}_{C}}{k_{R}^{C}+k_{F}^{C}\widetilde{B}_{C}}. \end{array} $$

Similarly, from Eqs. (30) and (33),

35 $$\begin{array}{@{}rcl@{}} \widetilde{L}_{N} = \frac{L_{N_{T}} k_{R}^{N}}{k_{R}^{N}+k_{F}^{N}\widetilde{B}_{N}} \text{,} \quad \widetilde{N}_{N} = \frac{L_{N_{T}} k_{F}^{N}\widetilde{B}_{N}}{k_{R}^{N}+k_{F}^{N}\widetilde{B}_{N}}. \end{array} $$

Substituting Eq. (35) into (27),

36 $$\begin{array}{@{}rcl@{}} \widetilde{B}_{N} = \frac{k_{\text{diff}}+k_{\text{active}}^{C}}{k_{\text{diff}} + k_{\text{active}}^{N}}\widetilde{B}_{C}. \end{array} $$

Substituting Eqs. (34) and (36) into (26),

37 $$\begin{array}{@{}rcl@{}} \widetilde{B}_{C} = \frac{B_{\text{synth}}}{k_{\text{deg}} \gamma \left(1 - \frac{W}{2}\right)}. \end{array} $$

Figure 8 shows the effect of *γ* on $\widetilde {N}_{C}(W)$ and $\widetilde {N}_{N}(W)$, where *W* represents the Wnt level in the cell. Decreasing *γ* increases the amount of bound adhesion complexes and bound transcription complexes.

### The effect of aPC mutation on models of crypt cells

Using the parameter values described in Tables 2 and 3, we can analyse the effect of mutations on the steady states for the VL model (given in (15)–(25) and Tan model (34)–(37)). Figure 8 shows the effect of varying expression of the APC mutation, *γ*, on the steady states of the bound adhesion and transcription complexes in the VL (*C*_*A*_ and *C*_*A*_) and Tan models (*N*_*C*_ and *N*_*N*_). Decreasing *γ* leads to more adhesion and transcription complexes, which in turn lead to an increased strength in intercellular adhesion between this cell and its neighbours, and to an increase in intracellular gene expression of target proteins responsible for cell-cycle control (and a resulting increase in proliferation and decrease in differentiation). In addition, in the VL model the results for the ‘apc1’ mutation shown here and the ‘mut2’ mutation considered in [30] (full results not shown here for brevity) are similar. Hence, for the VL model, reducing the rate of formation of destruction complexes and reducing the rate of *β*-catenin phosphorylation by the destruction complex have similar effects on adhesion and gene expression and therefore we only consider the ‘apc1’ mutation in this paper.

**Table 2:** Tan Parameter Values

*B*_synth_	1.306
*k*_deg_	0.0163
$k_{R}^{C}$	0.000647
$k_{F}^{C}$	0.00001
$k_{R}^{N}$	0.00349
$k_{F}^{N}$	0.0001
*k*_diff_	39.13
$k^{C}_{\text {active}}$	4.5
$k^{N}_{\text {active}}$	17.16
*V*_*C*_	1.16
*V*_*N*_	0.65

**Table 3:** Van Leeuwen Parameter Values

*s*_*A*_	20
*s*_*CA*_	250
*s*_*C*_	25
*s*_*CT*_	30
*s*_*D*_	100
*s*_*T*_	10
*s*_*X*_	10
*s*_*Y*_	10
*d*_*A*_	2
*d*_*CA*_	350
*d*_*C*_	1
*d*_*CT*_	750
*d*_*D*_	5
*d*_*Dx*_	5
*d*_*T*_	0.4
*d*_*u*_	50
*d*_*X*_	100
*d*_*Y*_	1
*K*_*C*_	200
*K*_*D*_	5
*K*_*T*_	50
*p*_*c*_	0
*p*_*u*_	100
*ξ*_*D*_	5
*ξ*_*Dx*_	5
*ξ*_*X*_	200
*ξ*_*c*_	0

### SBML-Chaste

The multicellular coupling described in this paper is implemented in a number of new C++ classes and python scripts which build upon the open source Chaste Library. SBML models were converted to a subcellular reaction network model (C++ class compatible with Chaste) which can be used within these multicellular simulations using a SBML translator developed for this project. The translator (written in Python) converts SBML to ODEs defined in C++. The translator uses libsbml [56] to parse the SBML and create the ordinary differential equations in the correct form for integration within a multicellular Chaste simulation.

All code required to run these simulations (including the SBML to Chaste translator) is available, along with user tutorials, under an open source licence at https://github.com/jmosborne/SBMLChaste.

Other SBML subcellular models of the Wnt pathway could be incorporated in the framework. This is done by incorporating the drag function (Eq. (3)) into the SBML model and specifying the appropriate threshold for nuclear *β*-Catenin for cells to progress through the cell cycle.

Both the Tan and Van Leeuwen subcellular systems, represented here as systems of ODES (26)-(31) and (4)-(14) respectively, are available as subcellular reaction networks in SBML (see Supplementary File 1 and 2 respectively).

## Supplementary information

**Additional file 1** **Supplementary Movie 1.** Video of simulation from Fig. 4. Snapshots from this movie are shown in Fig. 4. SupplementaryMovie1.mp4 or https://youtu.be/6scKT_JdUng.

**Additional file 2** **Supplementary Movie 2.** Video of simulation from Fig. 5. Snapshots from this movie are shown in Fig. 5. SupplementaryMovie2.mp4 or https://youtu.be/cd7kuL0tPOg.

**Additional file 3** **Supplementary Movie 3.** Video of simulation from Fig. 6. Snapshots from this movie are shown in Fig. 6. SupplementaryMovie3.mp4 or https://youtu.be/BVx0AZBlECc

**Additional file 4** **Supplementary file 1.** SBML for the VL model extended to include variable drag.

**Additional file 5** **Supplementary file 2.** SBML for the Tan model extended to include mutations and variable drag.

## Data Availability

All code required to run the simulations presented in this paper (including the SBML to Chaste translator) is available, along with user tutorials, under an open source licence at https://github.com/jmosborne/SBMLChaste.
